# Evidence of *In Vivo* Absorption of Lactate and Modulation of Short Chain Fatty Acid Absorption from the Reticulorumen of Non-Lactating Cattle Fed High Concentrate Diets

**DOI:** 10.1371/journal.pone.0164192

**Published:** 2016-10-07

**Authors:** Muhammad Qumar, Ratchaneewan Khiaosa-ard, Poulad Pourazad, Stefanie U. Wetzels, Fenja Klevenhusen, Wolfgang Kandler, Jörg R. Aschenbach, Qendrim Zebeli

**Affiliations:** 1 Institute of Animal Nutrition and Functional Plant Compounds, Department for Farm Animals and Veterinary Public Health, University of Veterinary Medicine Vienna, Vienna, Austria; 2 Institute for Milk Hygiene, Milk Technology and Food Science, Department for Farm Animals and Veterinary Public Health, University of Veterinary Medicine Vienna, Vienna, Austria; 3 Center for Analytical Chemistry, Department of Agrobiotechnology, IFA-Tulln, University of Natural Resources and Life Sciences in Vienna, Tulln, Austria; 4 Institute of Veterinary Physiology, Free University of Berlin, Berlin, Germany; National Institute for Agronomic Research, FRANCE

## Abstract

Short-chain fatty acids (SCFAs) and lactate are endproducts of rumen fermentation and important energy sources for the host ruminant. Because their rapid accumulation results in ruminal acidosis, enhancement of the absorption of SCFA and lactate across reticuloruminal wall is instrumental in increasing energy supply and preventing ruminal acidosis in cattle. This study investigated whether the reticuloruminal absorption of SCFAs and lactate was altered by different strategies of high concentrate feeding. Eight rumen-cannulated, non-lactating Holstein cows were fed a forage-only diet (baseline) and then gradually adapted over 6 d to a 60% concentrate level. Thereafter, this concentrate-rich diet was fed for 4 wk either continuously (Con; n = 8) or interruptedly (Int; n = 8). Absorption of SCFAs and lactate was determined in vivo from the experimental buffer introduced into the washed reticulorumen. The buffer contained acetate, propionate, butyrate and lactate at a concentration of 60, 30, 10 and 5 mmol/L, respectively and Cr-EDTA as a marker for correcting ruminal water fluxes. The reticuloruminal absorption after 35 and 65 min of buffer incubation was measured at the baseline, after 1 wk of 60% concentrate feeding in the interrupted model (Int-1) and after 4 wk of concentrate feeding in both feeding models (Int-4 and Con-4). Data showed that the absorption rates of individual and total SCFAs during the first 35 min of incubation of Con-4 were highest (~1.7 times compared to baseline), while Int-1 and Int-4 were similar to respective baseline. Lactate was not absorbed during forage-only baseline and 1-wk concentrate feeding, but after 4-wk feeding of concentrates in both models. In conclusion, SCFAs absorption across the reticulorumen of non-lactating cattle was enhanced by the 4-wk continuous concentrate feeding, which seems to be more advantageous in terms of rumen acidosis prevention compared to the interrupted feeding model. The study provides evidence of lactate absorption across the reticulorumen of non-lactating cattle after both continuous and interrupted 4-wk concentrate feeding.

## Introduction

Concentrates are important components in the diet of high-producing cattle, typically supplying easily fermentable carbohydrates as rapid energy source for rumen microbes and the host. The fermentation of easily fermentable carbohydrates results in the release of large amounts of short chain fatty acids (SCFAs) and, to a lesser extent, lactate in the ruminal fluid. These large amounts of SCFAs are absorbed across the ruminal epithelium enhancing the amount of metabolizable energy [1], needed to support high milk production or rapid growth rates. On the other hand, the absorption and rapid disappearance of luminal SCFAs and lactate is crucial for regulating ruminal pH within the physiological range [2]. When SCFAs production largely exceeds the absorption and neutralization capacity of the rumen, ruminal pH drops and can lead to subacute ruminal acidosis (SARA) [3]. The SARA is a prevalent metabolic disorder in cattle [4].

Despite the extended knowledge about causes and health consequences of high concentrate feeding and SARA in cattle [5], results regarding the association between concentrate-rich nutritional challenges and ruminal SCFAs absorption varied among studies [6, 7]. Furthermore, data for the ruminal capacity to absorb lactate in ruminants are controversial [8], likely depending on feeding conditions. For example, Harmon et al. [9] described that lactate was absorbed when steers were fed with a 70% concentrate diet, while Rowe et al. [10] reported no absorption of lactate from the caecal or rumen pouch. Indeed, epithelial adaptation affects SCFAs absorption, and its adaptive changes may depend on the duration and feeding pattern of the concentrate [11]. So far, to our knowledge, only a small number of studies were conducted to investigate ruminal absorption in response to high concentrate challenges in ruminants [7, 12]. Moreover, none has considered the effect of interrupted concentrate challenges with one or more weeks of a break from the high concentrate feeding challenge which however have been shown to increase the proton burden on the rumen [13, 14]. Previously, Dohme et al. [13] tested three consecutive single acidosis challenges and detected decreased ruminal pH profile (mean and nadir) with each challenge. They concluded that cows become more prone to acidosis over time despite the fact that they decrease grain intake to avoid acidosis. Likewise, Pourazad et al. [14] observed a greater severity of SARA in cows transiently induced with a high concentrate diet compared to those challenged continuously over some weeks. Accordingly we hypothesized that a continuous long-term concentrate challenge increases ruminal SCFAs absorption to a larger extent than an interrupted challenge. We investigated SCFAs and lactate absorption rates under isolated and washed reticulorumen conditions in cows fed a high concentrate diet for 4-wk either continuously or interruptedly.

## Materials and Methods

All procedures involving animal handling and treatment were approved by the institutional ethics committee of the University of Veterinary Medicine (Vetmeduni) Vienna and the national authority according to §26ff of the Law for Animal Experiments, Tierversuchsgesetz 2012- TVG (GZ 68.205/0093-II/3b/2013). The experiment was conducted at the university research farm Kremesberg of Vetmeduni Vienna, Austria. Non-pregnant non-lactating cows were used as the animal model in this study to avoid potential interference of differences in hormonal profile, lactation stages, feed and concentrate intake, as well as the complications due to intensive experimental measurements and procedures.

### Animals, Experimental Design, and Feeding

Experimental procedure and high concentrate challenge models were previously described in details by Pourazad et al. [14]. Briefly, the experiment was conducted with 8 rumen-cannulated (100 mm i.d.; Bar Diamond, Parma, ID) non-lactating Holstein cows (initial body weight (BW) and age: 710 ± 118 kg and 68 ± 20 months, respectively, mean ± SD) in a 2 × 2 crossover design (n = 8 per treatment). The experiment was conducted in two sequential runs with an 8-wk washout period in between. Cows were blocked by BW and randomly allocated to two concentrate challenge models which were one interrupted (Int) and another continuous (Con) model. At the start of the experiment, all cows received a forage-only diet consisting of 50% grass silage and 50% second-cut meadow hay (dry matter (DM) basis) for 2–3 wk. Next, there was a 6-d stepwise increment of the concentrate level (+10% daily DM basis) of the diet fed to all cows with the target of a diet containing 60% concentrate and 40% forage on DM basis (Table 1). Thereafter, cows in the continuous model remained on the 60% concentrate diet continuously for 4 wk. Cows in the interrupted model were fed the 60% concentrate diet for 1 wk followed by a 1-wk break by feeding the forage-only diet. Thereafter, the interruptedly-fed cows were re-challenged for another 2 wk with the first 2 d of a stepwise increment of concentrate intake. All cows were kept together in a loose-housing stable with straw bedding. They had access to feeder troughs which were equipped with electronic weighing scales and computer-regulated access gates (RIC system, Insentec B.V., Marknesse, The Netherlands). The forage and concentrate were offered in separate troughs. The separate feeding was meant to standardize the daily intake of concentrate for both challenge models. The distribution and feed intake of individual cows was monitored and recorded. All cows received fresh forage daily at 0800, followed by concentrate at 1000. During baseline and until d-4 of adaptation, diet was offered at 1.5% BW, whereas from d-5 of adaptation, the DM allowance was increased to 2% of BW. Fresh clean water and a mineral licking stone (RINDAMIN LECKSTEIN; Schaumann GmbH & Co KG, Brunn, Austria) were available to the animals throughout the experiment.

**Table 1:** Ingredients of forage-mix and concentrate and nutrient composition of the high concentrate diet.

Item	% of DM basis
Forage-mix composition^1^	
Grass silage	50.0
Second-cut meadow hay	50.0
Concentrate composition^2^	
Barley grain	33.0
Wheat	30.0
Corn	15.0
Rapeseed meal	17.0
Dried beet pulp	3.2
Calcium carbonate	0.5
NaCl	0.3
Mineral-vitamin premix^3^	1.0
SARA-challenge diet^4^	
DM (%)	74.5
OM	94.1
CP	15.4
NDF	31.8
ADF	19.9
Ash	5.86
Ether extract	1.71
NFC^5^	45.2

^1^The forage-mix consisted of (DM basis) 91.6% OM, 12.8% CP, 1.5% EE, and 51.7% NDF; DM content 54.4%.

^2^Concentrate contained (DM basis) 95.8% OM, 17.2% CP, 1.9% EE, and 19.5% NDF; DM content 88.0%.

^3^Mineral-vitamin premix contained (Schaumann GmbH & Co KG, Brunn, Austria, g/kg feed): Ca, 220; P, 60; Mg, 30; Na, 60; Zn, 3; Mn, 5; I, 0.01; Se, 0.04; Co, 0.03; Cu, 0.75; vitamin A, 600,000 IU; vitamin D, 80,000 IU; vitamin E, 2.

^4^Consisting of 40% forage-mix and 60% concentrate (DM basis).

^5^NFC = 100 –CP—NDF—Ether extract—Ash.

### Washed Reticulorumen Procedure

The temporarily isolated and washed reticulorumen procedure (WRP) was performed [15, 16] on all cows to determine the rate of SCFAs and lactate absorption across the reticulorumen epithelium. We performed WRP three times in the interrupted challenge model at baseline (Int-0, d-0), after 1-wk concentrate challenge (Int-1, d-14) and at the end of concentrate re-challenge (Int-4, d-35). In the continuous challenge model, WRP was performed at baseline (Con-0, d-0) and at the end of the concentrate challenge (Con-4, d-35). On the day of WRP, cows were restrained in a separate place with straw bedding on the floor. First of all, the reticulorumen contents were manually evacuated and stored in an insulated container kept in hot water to maintain the proper temperature of the digesta (38–39°C). Once emptied, the reticulorumen was washed three times with warm tap water (10 L/wash at 39°C) to remove the digesta particles attached to the reticulorumen wall, followed by 4 consecutive washings with washing buffer solution (10 L/wash) warmed at 39°C and with pH at 6.3–6.4 (Table 2). After each washing, the buffer solution was sucked out with a soft rubber tube connected to a vacuum pump.

**Table 2:** Composition of washing buffer and experimental buffer solutions (mmol/L) infused into the temporarily isolated and washed reticulorumen of cows.

Item	Washing buffer	Experimental buffer*
NaCl	120	10
NaHCO_3_	25	25
K2HPO_4_	0	5
CaCl_2_	0	2
MgCl_2_	0	2
Sodium acetate	0	60
Sodium propionate	10	30
Butyrate	0	10
Lactate	0	5
Cr-EDTA	0	1.8

*Gassed continuously with 100% CO_2_ during incubation in the rumen [15,16].

Subsequently, the reticulorumen was isolated from the rest of the gastrointestinal tract by occluding the esophagus with a nasogastric tube for saliva collection (University of Leipzig, Leipzig, Germany), and the omasal orifice with a Foley balloon catheter (Rusch Gold Foley Catheter 30–50 ml; Teleflex Medical., Athlore, Ireland). Saliva was continuously aspirated by a vacuum pump (N86KT45P; KNF Neuberger, Inc., Trenton, NJ) through the esophageal occluding device, collected in a glass bottle and the volume was noted. After the isolation of the reticulorumen, it was washed again with 10 L washing buffer solution to ensure no accumulation of saliva in the reticulorumen during the placement of esophageal occluding device. Afterwards, 20 L of experimental buffer solution (pH 6.6–6.7, preheated to 39°C, Table 2), were transferred into the reticulorumen and left in the reticulorumen precisely for 65 min. The experimental buffer solution was continuously gassed using 100% CO_2_ during the incubation time. The cannula was covered with a foam sponge lid, and gassing and sampling were done by tubes passing through this lid.

About 20 mL of the experimental buffer were collected at 0, 35 and 65 min after infusion and stored at -20°C for further analyses. The sample at 0 min was collected directly from the buffer container just before infusion into the reticulorumen. After 65 min, gassing was stopped, the experimental buffer was pumped out, the recovered volume was noted and then discarded. Subsequently, gassing tube and occluding devices were removed. Saliva collected during the WRP was poured back into the reticulorumen, rumen digesta was restored, and the cannula was closed.

### Analysis of Chromium, SCFAs, and Lactate

Chromium concentrations of buffer samples were measured using atomic absorption spectrometry at 357.9 nm wavelength. The platform atomization from a transversally heated pyrolytically coated graphite tube was used. Argon was used as purge gas. Background correction was performed by longitudinal Zeeman effect (4100 ZL Spectrometer with AS-70 autosampler; PerkinElmer Inc., Waltham, MA). The 100 μL of the experimental buffer samples were diluted to 50 mL (1:500) with 0.5% nitric acid prior to measurement. Calibration standards contained 0, 20, 40, 100 and 200 μg/L Cr in 0.5% nitric acid. Five μL sample or standard were injected together with 13 μg Mg(NO_3_)_2_ in 15 μL 0.5% nitric acid as matrix modifier. Pyrolysis temperature was 1,300°C and atomization temperature 2,300°C. Sensitivity was checked by analysis of certified reference material NIST 1640a (Trace Elements in Natural Water, Standard Reference Material^®^, Gaithersburg, MD) after each calibration and by repeated measurement of the standard (100 μg/L) every 8 samples.

Short chain fatty acid concentration (acetate, propionate, and butyrate) was determined by gas chromatography. Experimental buffer samples were thawed and then centrifuged at 20,000 × *g* for 25 min at 4°C to clear the buffer from traces of digesta particles prior to analysis. An external standard mixture (acetate: propionate: butyrate at 3:2:1 v/v/v, Sigma-Aldrich Co. LLC. St Louis, MI) or the supernatant (0.6 mL) was transferred into a fresh tube and 0.2 mL of HCl (1.8 mol/L) was added, followed by 0.2 mL of the internal standard (4-methylvaleric acid, Sigma-Aldrich Co. LLC. St Louis, MI). The mixture was centrifuged at 20,000 × *g* for 25 min at 4°C to remove any precipitated substrates. The clear supernatant was transferred into the GC vial and was analyzed for SCFAs concentrations via gas chromatography apparatus (GC Model 8060 MS 172 DPFC, No.: 950713, Fisons, Rodena, Italy) which was equipped with a flame-ionization detector and a 30 m × 0.53 mm ID × 0.53μm df capillary column (Trace TR Wax, Thermo Fisher Scientific, Waltham, MA). Injector and detector had temperatures of 170°C and 190°C. Helium was used as carrier gas with a flow rate of 1 mL/min. Stratos Software (Stratos Version 4.5.0.0, Polymer Laboratories, Church Stretton, Shropshire, UK) was utilized for generation and evaluation of chromatograms.

Concentrations of total lactate were determined by the oxidation of lactate to pyruvate in the presence of NAD^+^ using a commercially available kit (Megazyme D-, L-lactate assay kit; Megazyme international, Wicklow, Ireland). In short, non-deproteinized buffer samples were mixed with the assay buffer containing D-glutamate, NAD^+^, and D-glutamate-pyruvate transaminase. Then, lactate dehydrogenase was added to the mixture. After the reaction, the absorbance was read at 340 nm wavelength with microplate reader (BIO-RAD Laboratories GmbH, Munich, Germany).

### Calculations of SCFAs and Lactate Absorption Rates

Chromium is a common marker used in previous WRP studies [17–19] as it is assumed that Cr is not absorbed through the rumen. Chromium concentration was therefore used to estimate total buffer volume which was then used for calculating the acid pool (mmol). Subsequently, absolute absorption rate (mmol/h) and fractional rate (%/h) were calculated as follows:

$$\text{mmol}/\text{h }=\;\frac{S1-S2}{T2-T1}\times 60$$

$$\%/\text{h }=\;\frac{\frac{S1-S2}{T2-T1}\times 60}{V1}\;\times 100$$

Where S1 and S2 are the initial and final amounts of analyte in 20 L of experimental buffer solution, respectively, T1 is the initial time point, and T2 is the final time point (min). Data of the formulated contents of SCFAs, lactate, and Cr as well as the analyzed data in the initial buffer are shown in Table 3.

**Table 3:** Composition of Cr, SCFAs, and lactate of 0-min experimental buffer solution.

Item	Formulated	Observed	
		Mean ± SD	Median
Cr (mmol/L)	1.80	1.78 ± 0.105	1.81
Concentration (mmol/L)			
Acetate	60.0	63.9 ± 4.92	63.21
Propionate	30.0	32.1 ± 2.76	32.18
Butyrate	10.0	10.4 ± 0.95	10.17
Total SCFAs	100.0	106.3 ± 8.20	105.8
Lactate	5.0	6.5 ± 0.92	6.57
Composition (% of total SCFAs)			
Acetate	60.0	60.11 ± 0.78	60.05
Propionate	30.0	30.13 ± 0.82	30.24
Butyrate	10.0	9.76 ± 0.61	9.64

### Statistical Analysis

All statistical analyses of the data were performed using the PROC MIXED of SAS (version 9.4; SAS Inst. Inc., Cary, NC). Means ± SD and medians of Cr concentration, SCFAs concentration and composition of 0-min experimental buffer were generated using PROC MEANS of SAS. Data were tested for normality by Shapiro-Wilk test using the PROC UNIVARIATE of SAS. Because of unequal WRP measurements between both concentrate challenge models, thus in order to compare all challenge periods, we considered Int-0, Int-1, Int-4, Con-0 and Con-4 as independent periods and therefore the data were analyzed as a complete randomized block design. The statistical model consisted of fixed effects of challenge period and the experimental run and random effects of cows within experimental run. The linear mixed model was:

$$Y_{ijk}=\;\mu \;+\;A_{i}+\;B_{j}+\;C_{k(j)}+\;e_{ijk};$$

Where Y is the dependent variable, μ is the overall mean, A is the fixed effect of challenge period, B_j_ is the fixed effect of experimental run, C is the random effect of animals within run, and e is the residual error. For all analyses, degrees of freedom were estimated using the Kenward-Roger method. The values are reported as least square means and their multiple comparisons between feeding periods were obtained from the CONTRAST analysis. The significance level was set at *P* ≤ 0.05, and a trend was considered up to the 0.05 < *P* ≤ 0.10 level.

## Results

### Dry Matter Intake

As intended, the DMI of cows was similar during baseline feeding between the two feeding challenge strategies (11.0 and 11.4 kg/d for Int-0 and Con-0 cows, respectively, *P* > 0.05) and compared to the baseline the DMI increased (*P* < 0.05) during high concentrate periods (14.9, 15.3 and 16.3 kg/d for Int-1, Int-4 and Con-4, respectively; SEM = 0.68: *P* < 0.05 only for Int-1 vs. Con-4). The similar DMI between the two feeding challenge strategies in baseline and in high concentrate periods was meant to elucidate the underlying causes for the ruminal changes in response to the diet challenge beyond of the quantity of the substrates for ruminal fermentation.

### Absolute Absorption Rates of SCFAs and Lactate

Based on the estimation using Cr, the absolute absorptions of SCFAs (mmol/h) after 0–35 and 35–65 min of incubation are shown in Table 4. All values of both Int-0 and Con-0 were similar (*P* > 0.05). The effect of continuous vs. interrupted challenge periods on individual and total SCFAs was found only for 0–35 min of incubation by which Con-4 showed the highest value among all treatments and thus it was 1.7 times higher than Con-0 (*P* < 0.05). Total SCFAs was absorbed in 0–35 min of incubation at the rate of 883 mmol/h in Con-4. Acetate, propionate and butyrate contributed with 476, 305 and 102 mmol/h to total SCFAs absorption, respectively. By contrast, Int-1 and Int-4 failed to show such improvement as their absorption rates were approximately 0.96 and 1.17 times Int-0, respectively. For the last 30 min of incubation, all challenge periods had similar values.

**Table 4:** Absolute rate of absorption (mmol/h) of SCFAs determined by the washed reticulorumen procedure as affected by high concentrate challenge model^1^ (interrupted model (Int) and continuous model (Con)) measured before (Int-0 and Con-0) and after 1 wk (Int-1) and 4 wk (Int-4 and Con-4) of challenge.

Item	Challenge period					SEM	*P* value^2^		
	Int-0	Int-1	Int-4	Con-0	Con-4		Int-0 vs Int-1	Int-0 vs Int-4	Con-0 vs Con-4
Total SCFAs									
0–35 min	536	518	627	501	883	120.1	0.916	0.605	0.031
35–65 min	780	514	770	686	671	140.1	0.126	0.952	0.930
Acetate									
0–35 min	284	281	335	270	476	69.3	0.981	0.617	0.042
35–65 min	442	276	425	382	372	86.5	0.120	0.858	0.921
Propionate									
0–35 min	187	178	213	169	305	39.5	0.867	0.656	0.020
35–65 min	256	180	261	231	227	46.6	0.128	0.927	0.936
Butyrate									
0–35 min	65	59	79	63	102	12.1	0.721	0.420	0.026
35–65 min	81	58	85	73	72	13.1	0.178	0.812	0.966
Lactate									
0–35 min	-26	-33	-4	-57	-20	16.2	0.777	0.356	0.109
35–65 min	-39	-10	16	-25	36	16.5	0.214	0.026	0.012

^1^Cows were fed a 60% concentrate diet (DM basis) for 4 wk, either continuously (Con) or with a 1-wk interruption (forage-only) in the second wk of the challenge period (Int).

^2^Contrast analysis

The absolute rates of lactate absorption (mmol/h) are shown in Table 4. There was no apparent absorption of lactate during Int-0 and Con-0 as the estimated values were less than zero. However, the high concentrate challenge increased the absorption of lactate, but, unlike SCFAs, this was evident during 35–65 min of incubation, while there was no challenge period effect for 0–35 min of incubation. Both Int-4 and Con-4 had higher lactate absorption values (16.3 mmol/h and 36.2 mmol/h respectively) than their baseline values (*P <* 0.05), while Int-1 was not different from Int-0.

### Fractional Absorption Rates of SCFAs and Lactate

An effect of challenge period on fractional absorption rates of individual and total SCFAs was also exclusively noted for 0–35 min of incubation (Table 5). Again, Con-4 exhibited the highest fractional absorption rate of total SCFAs (40%) and each individual SCFA (36–48%/h depending on SCFAs). All values accounted for approximately 1.7 times Con-0 (*P* < 0.05). In contrast, Int-1 and Int-4 had similar fractional absorption rates compared to Int-0. Differences among challenge periods were not found for the fractional absorption rates of total or individual SCFAs for 35–65 min of incubation.

**Table 5:** Fractional absorption rate of (%/h) of SCFAs determined by the washed reticulorumen procedure as affected by high concentrate challenge model^1^ (interrupted model (Int) and continuous model (Con)) measured before (Int-0 and Con-0) and after 1 wk (Int-1) and 4 wk (Int-4 and Con-4) of challenge.

Item	Challenge period					SEM	*P* value^2^		
	Int-0	Int-1	Int-4	Con-0	Con-4		Int-0 vs Int-1	Int-0 vs Int-4	Con-0 vs Con-4
Total SCFAs									
0–35 min	25.7	24.3	28.6	23.7	40.1	5.32	0.850	0.708	0.037
35–65 min	39.9	29.1	40.4	37.3	40.2	6.14	0.147	0.937	0.698
Acetate									
0–35 min	22.6	21.8	25.6	21.3	36.0	5.14	0.911	0.698	0.051
35–65 min	36.8	25.3	36.1	34.0	35.6	6.17	0.127	0.927	0.833
Propionate									
0–35 min	29.6	27.9	32.4	26.3	45.5	5.74	0.830	0.739	0.024
35–65 min	44.5	35.3	47.4	42.3	47.6	6.20	0.212	0.675	0.472
Butyrate									
0–35 min	32.5	28.5	35.2	30.5	48.0	5.35	0.596	0.735	0.027
35–65 min	45.5	34.4	46.4	43.1	48.2	6.27	0.156	0.903	0.514
Lactate									
0–35 min	-17.9	-30.6	-5.4	-47.4	-18.0	13.01	0.490	0.510	0.115
35–65 min	-29.0	-11.6	12.9	-17.7	25.0	11.92	0.301	0.020	0.015

^1^Cows were fed a 60% concentrate diet (DM basis) for 4 wk, either continuously (Con) or with a 1-wk interruption (forage-only) in the second wk of the challenge period (Int).

^2^Contrast analysis

Fractional absorption rates of lactate are presented in Table 5. Consistent with results of absolute absorption rates, fractional absorption rates during baseline were below zero. An influence of concentrate challenge period on the fractional absorption rate was apparent for 35–65 min of incubation. Here, only Int-4 and Con-4 but not Int-1 showed an increase in fractional absorption rate of lactate compared with the corresponding Int-0 and Con-0 (*P* < 0.05).

## Discussion

To date, most of the studies assessed SCFAs absorption and rumen epithelial adaptation based on single and continuous high concentrate challenge patterns in an attempt to induce SARA [7, 20, 21]. In fact, SARA conditions may fluctuate since cows would try to reduce feed intake especially of grains to restore the ruminal homeostasis [13], but the knowledge about intermittent bouts of transient SARA is very limited. Increasing severity of SARA after repeated challenges was reported previously [13]. In line with that, our companion study [14] showed that both interrupted and continuous concentrate challenge models had a similar pH profile during the first wk of challenge (220–318 min per day at pH < 5.8), but after re-challenge the severity of SARA was more pronounced in interruptedly fed cows (on average 500 min per day) than in continuously fed ones (278 min per day). The present study now shows that the ruminal pH profile at the end of interrupted vs. continuous challenges described in our companion study [14] may be explained, to some extent, by altered SCFAs absorption. SCFAs absorption was lower for animals experiencing more severe SARA at the end of the interrupted adaptation (Int-4) than for animals experiencing only moderate SARA at the end of continuous adaptation (Cont-4). This is in line with previous conclusions that ruminal SCFAs absorption is the key determinant of intraruminal pH and the susceptibility of SARA [6]. Obviously, an adaptation of acid-absorptive capacity over several weeks of a dietary adaptation to high concentrate diet is crucial to keep pace with an increasing requirement for acid absorption. Any interruption of this adaptation to concentrate feeding may largely impact on SARA incidence.

The increasing absorptive capability of the ruminal epithelium with increasing length of a concentrate challenge was already reported by [7] who observed higher SCFAs absorption in cows that were fed a high grain diet for a longer time than those cows fed the same diet but for a shorter period of time. The underlying epithelial adaptation can be either functional or morphological [2]. Both the concentration and composition of SCFAs in the rumen might be able to impose a challenge to the ruminal epithelium, leading to a stimulation of the adaptive response. Supporting this notion, there is evidence for effects of high energy diets on promoting morphological adaptation like cellular proliferation [22] and functional adaptation [12]. Previously, it has been described that the fractional rate of SCFAs absorption increased due to a comprehensive increase of the surface area of the ruminal epithelium with increased luminal availability of butyrate [23]. Because such morphological adaptation may require a total duration of up to 6 or 8 wk [11, 24], morphological changes may possibly explain that a longer adaptation in the continuous challenge model induced a higher absorptive capacity for SCFAs.

Absorption of SCFAs is dependent on both apical uptake from the rumen and basolateral release into the blood stream. Apical uptake can be in the undissociated form by lipophilic diffusion or in the dissociated form by anion transport proteins [25]. Because a buffer pH of 6.7 was used in our WRP, the vast majority of SCFAs should be in the dissociated form and thus there was a significant participation of anion-transport proteins in SCFAs absorption, particularly, for acetate which is the least lipophilic SCFAs [3]. Commonly, apical uptake is considered the rate limiting step of ruminal SCFAs absorption. However, removal of SCFAs from the serosal side of the epithelium by blood controls the net transepithelial flux of SCFAs and, thereby, determines SCFAs absorption. Finally, it must be acknowledged that ruminal microbiota, ruminal passage rate and other factors may also interact with ruminal SCFAs absorption but their discussion would be beyond the scope and data structure of the present study.

The fact that SCFAs absorption was not increased during Int-1 (i.e., 1-wk continuous challenge after baseline) and Int-4 (i.e., 2-wk continuous challenge after break) compared to respective baseline (i.e., Int-0), indicates that 1–2 wk of the high-concentrate feeding was not sufficient to promote significantly increased SCFAs absorption. This is in contrast to some previous studies where SCFAs and Na^+^ absorption measured in vitro increased already after 1 wk of transition to a highly fermentable diet [12, 26]. The improvements in Na^+^ and acetate absorptions occurred transiently, while the increased butyrate absorption persisted in response to the dietary transition in one of those studies [12]. Nonetheless, it must be acknowledged that a discrepancy among studies exists concerning ruminal epithelium adaptation in response to the increased exposure to luminal SCFAs [27, 28]. It was indicated that the ruminal adaptive response to increasing SCFA load was more pronounced in periparturient dairy cows than in cows with several weeks into lactation [28]. Also, non-lactating and non-pregnant cows seemed to be less responsive for acetate but more responsive for propionate and butyrate absorption during high grain feeding [6]. Since we worked with non-lactating and non-pregnant cattle in the present study, the unresponsiveness of SCFA absorption to short bouts of SARA during Int-1 and Int-4 appears, at least, congruent with the latter study [6].

There is inconsistent information in the literature regarding lactate absorption in the forestomach of ruminants. While some showed no absorption [10], others indicated the opposite [9, 29, 30]. The present study apparently resolves this controversy by showing that lactate can be absorbed but this requires triggering by a high grain diet for, at least, 2 wk (i.e., increased lactate absorption detected in Int-4). Previous studies suggested that lactate absorption from the rumen is mediated through monocarboxylate transporters (MCT); luminal uptake of lactate occurs through apical MCT4 while basolateral MCT1 transports lactate towards the blood [31, 32]. Coherent with a requirement of induction of lactate absorption by rumen metabolites during high grain feeding, [25, 33] clearly demonstrated that activity of apical MCT is extremely low in roughage fed sheep and goats. However, ruminal butyrate infusions in vivo stimulated MCT4 expression [23], whereas the incubation of isolated ruminal epithelia with butyrate ex vivo stimulated both MCT4 and MCT1 expression [34], the same as did the feeding of 60% grain on DM basis in the rumen and colon in goats [33]. Furthermore, low ruminal pH is known to increase the production rates of conjugated linoleic acids [35] which, in turn, may increase MCT1 activity in the ruminal epithelium via PPAR-α signaling [36]. This strongly suggests that metabolites released from ruminal fermentation as well as biohydrogenation may induce an ability to absorb lactate; even though lactate concentrations are mostly very low in events of SARA due to its rapid utilization by ruminal bacteria [37]. The low gradient of lactate in the rumen may therefore decrease significance of lactate absorption in comparison to SCFAs. Nonetheless, the induction of an ability to absorb lactate may be highly beneficial under high concentrate feeding, because counteraction of lactate accumulation in the rumen can protect the animal from drifting into life-threatening acute acidosis [3]. From this perspective, it is important to note that induction of lactate absorption was already efficiently induced by the interrupted challenge, which underlines that nature has given relatively high priority to this adaptation.

Interestingly, unlike SCFAs, the challenge showed an effect on lactate absorption for the later incubation time (i.e., 35–65 min of incubation). The reason behind this is not completely clear. One explanation could be that the high concentration of SCFAs in the luminal solution was interfering with lactate uptake via apical MCT from 0–35 min; and only after SCFAs concentration had been effectively lowered by ~50%, lactate absorption became measurable in the second period from 35–65 min.

The MCT is a bidirectional transporter and, similar to previous studies [7, 10], we experienced a number of minus absorption rates (i.e., higher lactate concentration at advancing incubation time). Previously, [7] described this as the net secretion, presumably as a result of the saturation of reticulorumen epithelium due to the intraepithelial metabolism of propionate [10]. Alternatively, such lactate secretion could also derive from the metabolism of glucose taken up from the blood stream.

## Conclusions

It can be concluded that feeding of 60% concentrate in the diet DM promoted ruminal SCFAs absorption in cattle. However, the feeding pattern played a key role. Compared with forage-only baseline feeding, the continuous concentrate feeding pattern over 4 wk exerted a positive effect on SCFAs absorption across the rumen wall, while the interrupted condition with a 1 wk of concentrate break between two shorter challenge periods did not change ruminal absorption capability. The enhancement in SCFAs absorption could partly elucidate the lesser severity of SARA with the persistent condition reported earlier. In addition, the present study proclaims that adaptation of the reticulorumen epithelium, as a mechanism to increase the SCFAs absorption capability, needs more than 1–2 wk in response to feeding of highly fermentable diets in cattle and an interruption of the feeding pattern could stall the adaptation process of the rumen epithelium. Finally, the study provides evidence that lactate can be absorbed through the rumen which was induced by both 2 wk of the re-challenge (interrupted challenge) and 4 wk of the continuous challenge.

## Supporting Information

S1 Data Data for absorption rates of short chain fatty acids and lactate (XLSX)
